# Framework Development of Non-Face-to-Face Training of Basic Life Support for Laypersons: A Multi-Method Study

**DOI:** 10.3390/healthcare11142110

**Published:** 2023-07-24

**Authors:** Sangsoo Han, Choung Ah Lee, Won Jung Jeong, JuOk Park, Hang A Park

**Affiliations:** 1 Department of Emergency Medicine, Soonchunhyang University Bucheon Hospital, 170 Jomaru-ro, Bucheon 14584, Republic of Korea; brayden0819@daum.net; 2 Department of Emergency Medicine, Hallym University, Dongtan Sacred Heart Hospital, Hwaseong 14068, Republic of Korea; juok.park@gmail.com (J.P.); hangapark@hallym.or.kr (H.A.P.); 3 Department of Emergency Medicine, Catholic University of Korea, St. Vincent’s Hospital, Suwon 16247, Republic of Korea; medpooh@naver.com

**Keywords:** education, cardiopulmonary resuscitation, face-to-face training, non-face-to-face training, Delphi

## Abstract

The spread of infectious diseases has accelerated the transition from face-to-face (F2F) to non-F2F (NF2F) education. To maintain the effect of successful NF2F education in cardiopulmonary resuscitation, reorganizing the curriculum to suit the NF2F educational environment is necessary. We propose an appropriate learning curriculum for NF2F basic life support (BLS) training for laypersons based on expert surveys and learners’ performance outcomes. This study included three stages and used multiple methods. A draft curriculum was created through a literature review and three-round Delphi approach, and then applied as a test for actual education. After the training, the final curriculum of the NF2F BLS training for laypersons was proposed by reflecting on the performance outcomes of learners and expert opinions. NF2F theoretical education was simplified into five content items: concept of chain of survival, legal protection for first aiders, importance of bystander cardiopulmonary resuscitation, how to recognize a patient in cardiac arrest and activate the emergency medical services system, and reduced training time. In the hands-on skills session, it was recommended to practice chest compressions using a simple intuitive feedback device and to use automated external defibrillators step-by-step more than in F2F training. In conclusion, NF2F training is a suitable option for BLS training methods in situations where F2F training is difficult.

## 1. Introduction

Traditional education has long been based on a teacher-centered approach using face-to-face (F2F) training. However, this has changed to a form based on communication between learners and teachers that is non-F2F (NF2F), such as online, distance, or electronic learning (e-learning) [1]. As the risk of infection owing to the coronavirus disease 2019 (COVID-19) pandemic increased, opportunities to provide education decreased [2]. Accordingly, development of NF2F learning, such as distance learning, e-learning, and blended learning, as a new education method to maintain the continuity of education, was accelerated [3]. This change in teaching methods also had an impact on basic life support (BLS) training. The European Resuscitation Council recommended discontinuing F2F BLS training for the general public in response to the COVID-19 pandemic and highlighted the relevance of NF2F learning to minimize the risk of infection transmission [4].

In situations where there is no direct cardiopulmonary resuscitation (CPR) training, the distance-learning method has the advantages of cost-effectiveness, time savings, learning autonomy and flexibility, and standardization of the delivery of training content, and it is better than no training [5]. Furthermore, the distance-learning method for laypersons showed noninferiority in chest compression depth, rate, complete recoil, and composite chest compression score compared with the traditional method [5,6,7].

However, NF2F education has some limitations. In NF2F education, it is difficult to focus on education, the quality of interaction and feedback is low, and the communication environment must be equipped with technical devices [8]. The curriculum and teaching methodologies that are successful in F2F learning are not always successful in NF2F education. In particular, in the field of CPR education, the translation of skills acquired in the classroom to a real clinical environment is emphasized [9]. In light of its heavy reliance on physical interactions and immediate technical feedback between teachers and learners, the acquisition of practical skills, including CPR, has been commonly advised to take place through F2F education rather than NF2F education [10]. For effective NF2F learning, it is necessary to reorganize the training content and methodological procedures. Several studies have compared BLS skills attained by applying new learning methods, but the learning content and composition have not yet been evaluated.

Therefore, the purpose of this study was to propose an appropriate learning curriculum for NF2F BLS training for laypersons on the basis of expert surveys and learners’ performance outcomes.

## 2. Materials and Methods

**Figure 1:**
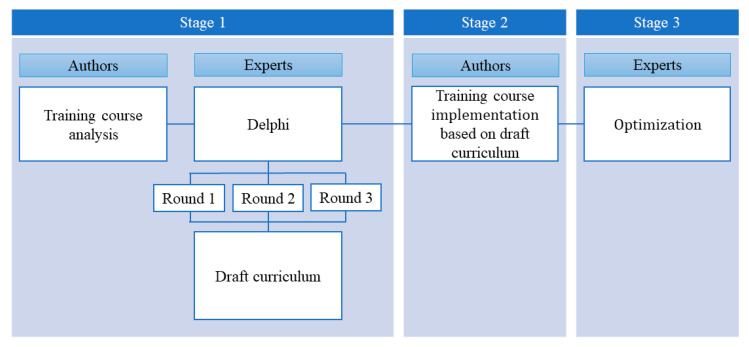
Summary of methods.

This study was divided into three stages and used multiple methods [11]. In the first stage, a draft curriculum for NF2F learning was created by analyzing the contents of the current training course and reflecting upon expert surveys. In the second stage, the newly drafted curriculum was implemented using an actual NF2F education method, and the effect was evaluated. Lastly, the curriculum was optimized on the basis of learners’ performance and expert opinions (Figure 1). This study was approved by the institutional review board of Hallym University (HDT 2020-06-023), and all participants provided informed consent.

### 2.1. Stage 1: Draft Curriculum for NF2F BLS Learning

Four steps (questionnaire preparation, expert panel, survey progress, and Delphi results) were performed [12].

#### 2.1.1. Standard CPR Training Course Analysis: Questionnaire Preparation

The standard BLS training course is based on video content that is typically a mixture of theoretical and hands-on skills training sessions in the form of practice while watching the video. The authors organized the core contents presented in the CPR guidelines [13] and classified them into theoretical and hands-on skill parts according to the implementation method. A questionnaire for all rounds was designed to assess the feasibility and effectiveness of the NF2F training operation for each content (Table S1). Questions were asked with responses scored on a five-point Likert scale: strongly disagree (1), disagree (2), neither agree nor disagree (3), agree (4), and strongly agree (5). Additionally, questions were asked regarding the necessity for shortening the training time and simplifying the operation in the first round. In the final round, content deemed unnecessary was selected in a plural fashion. Additionally, open-ended questions regarding considerations in NF2F education were provided.

#### 2.1.2. Expert Panel Identification and Recruitment

A criterion-based convenience sampling method was used to identify the panel of experts who participated in this study. The members of the Education Task Force Committee and BLS Taskforce Committee in the Korean Association of Cardiopulmonary Resuscitation were selected to form a panel of experts. A personalized invitation letter describing the background and purpose of the study, along with a consent form, was sent by email to 20 professional experts. Seven experts declined to participate because they had no experience in NF2F education, and 13 experts confirmed their willingness to participate in this study.

#### 2.1.3. Delphi Survey

A three-round modified Delphi approach was implemented with online questionnaires, from December 2020 to March 2021 [14]. The response period in every round was 2 weeks, and a reminder text message was sent to experts who did not complete the questionnaires within 5 days. In the second and third rounds, the same questionnaire as that used in round 1, along with a summary of expert opinions from the previous round, was distributed to the participants.

#### 2.1.4. Establishing Consensus: Delphi Results

Percentage agreement was used to determine the consensus [15]. If at least 70% of the experts rated an item as 4 or 5, we defined that item as agreed upon by experts. Items were removed from the curriculum if more than 70% of experts judged the content as unnecessary.

### 2.2. Stage 2: Application of Draft Curriculum in NF2F Training

NF2F education was implemented using the draft curriculum. Among the NF2F education methods, a self-learning method with e-learning was selected, and its performance was compared with that of the F2F education method.

#### 2.2.1. Sample Size

The sample size calculation was conducted in a previous study [16]. Presuming an exclusion rate of 25%, a sample size of 70 participants per group was required to have 80% statistical power assuming an α-value of 0.05.

#### 2.2.2. Recruitment of Participants

To minimize bias due to technical difficulties, high-school students who were proficient in using smart devices were considered for enrolment. Participants were recruited through announcements on the BLS training site from July 2021 to April 2022. Participants were excluded if they had physical or communication disabilities during CPR training. They were randomly assigned in a 1:1 ratio to the F2F or NF2F method using randomly permuted blocks of size 2.

#### 2.2.3. Intervention

In the F2F group, training involved a video being shown on site by an instructor. In the NF2F group, each participant was provided with a mannequin and tablets in an isolated place. The assignment function of Kahoot! (Oslo, Norway) was used for the NF2F learning. Among the original educational videos, the clips of the adopted educational steps were separated and arranged in a predetermined order. Upon completion of the previous step, the learners were allowed to select the next step. The Innosonian Brayden Pro^®^ mannequin (Seoul, Republic of Korea) was used for visual feedback. The total training time was 40 min, and the training sessions were video-recorded.

#### 2.2.4. Measurements

**Figure 2:**
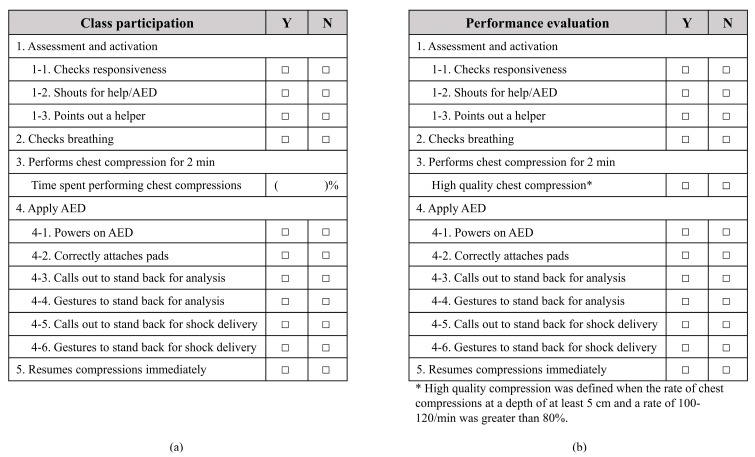
Class participation checklist for each step (**a**) and post-training evaluation checklist (**b**). AED, automatic external defibrillator.

The checklist for assessment of class participation and performance after education was developed on the basis of the adult skills testing checklist of the American Heart Association. The evaluation criterion pertaining to breathing was excluded, and, to emphasize safe defibrillation, analysis and shock delivery processes were evaluated by separating voice notification and gesture (Figure 2). To assess the degree of emersion in the training, we evaluated whether the participants had followed the hands-on sessions from the video. In the 2 min chest compression session, during the 2 min sample video, the duration (s) that chest compressions were performed following the video was determined and converted into a percentage. Post-training evaluation was conducted in an F2F environment for accurate verification. Whether each step was performed was evaluated, and the chest compression component was specifically evaluated to determine whether high-quality compression was maintained. High-quality compression was defined when the rate of chest compressions at a depth of at least 5 cm and the rate of 100–120 compressions/min exceeded 80%. The depth and rate of chest compression were measured using Laerdal Resusci Anne QCPR^®^ manikins (Stavanger, Norway).

#### 2.2.5. Data Analysis

Categorical variables are presented as numbers and percentages, and continuous variables are presented as medians and interquartile ranges. Categorical variables were compared using the chi-square test. Nonparametric continuous variables were analyzed using the Mann–Whitney U test. Statistical significance was set at a *p*-value < 0.05. All statistical analyses were performed using the SPSS software (version 25.0; IBM Corp., Armonk, NY, USA).

### 2.3. Stage 3: Development of Curriculum in NF2F BLS Training

The results of training using the draft curriculum were shared with 13 experts who participated in the earlier Delphi rounds. In the Delphi, they were requested to suggest improvements in content that showed low feasibility and efficiency, and low class participation and performance. Accordingly, the NF2F BLS training curriculum was finalized.

## 3. Results

### 3.1. Stage 1: Establishment of a Draft BLS Curriculum for Laypersons

The median age of the 13 experts who participated in the core content evaluation was 48 years, and 69.2% were males. The median BLS instructor experience of the experts was 15 years, and they had participated in 30 training sessions in the last 2 years (Table 1). Table 2 summarizes the feasibility and effectiveness of the NF2F training operation for each element of the final round.

**Figure 3:**
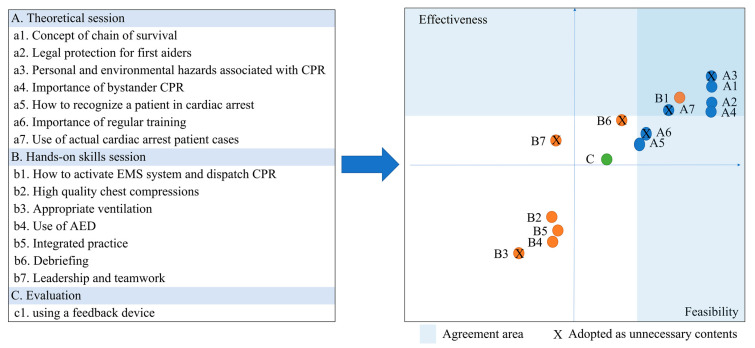
Core content reconstructed according to education method and results of Delphi rounds. AED, automatic external defibrillator; CRP, cardiopulmonary resuscitation; EMS, emergency medical services.

All experts agreed on the necessity for shortening the training time and simplifying its implementation for NF2F learning. To shorten the training time, contents deemed less necessary were removed. In the hands-on skills session, it was finally decided to delete the ventilation, debriefing, and teamwork/leadership items, as it was considered that the hands-only compression should be emphasized more in the NF2F situation (Tables S2 and S3). Debriefing and teamwork/leadership items were deleted because it was considered difficult to maintain interactive relationships in the NF2F education (Figure 3). The course order was rearranged from the method, in which theoretical and hands-on skills sessions were cross-implemented to a form, with which the theoretical session was completed, and after which hands-on skills sessions were conducted. Table 2 presents the draft curriculum.

Figure 3 shows the core content list according to the education method and Delphi results for each content item’s effectiveness, feasibility, and unnecessariness.

### 3.2. Stage 2: Application of Draft Curriculum in NF2F Training

There were no significant differences in terms of sex, age, or previous BLS training history between the participants in the F2F and NF2F groups (Table 3).

Class participation of the two groups showed significant differences except for the steps of checks for responsiveness, turning on of the automatic external defibrillator (AED), and attaching the pads. Within the NF2F learning group, class participation was remarkably low but was improved in the performance test for the analysis stage using call for help/AED (31.5% vs. 82.9%), identifying a helper (34.3% vs. 68.6%), calling out to stand back (20.0% vs. 28.6%), and gesturing to stand back for analysis (28.6% vs. 37.1%). Nevertheless, regarding the performance elements, checking breathing (31.4% vs. 28.6%), correct attachment of the AED pads (94.4% vs. 74.3%), and gesturing to stand back for shock delivery (37.1% vs. 28.6%) were worse. In terms of the high-quality chest compression steps, the two groups did not differ in terms of class participation and post-educational performance evaluation (Table 4).

### 3.3. Stage 3: Final Conceptualisation

The overall evaluation after the training operation was as follows: during NF2F skill practice, learners showed low participation in most steps, except for response checks and chest compressions. In the post-training evaluation, compared with that of the participants of F2F learning, performance was low in those who participated in NF2F learning, but the difference decreased. In particular, except for the step of turning on power of the AED, the performance related to the use of AED was significantly reduced in the post-training evaluation.

The results of the NF2F training were shared with 13 experts who participated in the Delphi, and opinions were collected for education improvement. When asked regarding the need for improvement in areas where participation in education dropped compared with the results of the post-training evaluation, eight experts (61.5%) suggested that the existing method could be maintained. They mentioned that environments other than classrooms can lead to more distractions, and simulated isolation rooms in video-monitored situations can discourage students from participating in classes. For items with low achievement, it was suggested to reinforce theoretical education through repetitive learning, use of smart devices, and practice. Regarding the use of feedback equipment during training, 11 experts (84.6%) answered that increasing trainees’ fatigue due to equipment settings should be avoided, and that feedback equipment should be simple to use even if it does not provide accurate figures. Regarding evaluation using a feedback device, which received a low score in feasibility and effectiveness in the Delphi rounds, 11 experts (84.6%) answered that the use of a feedback device is essential, even if it is inconvenient to operate (Table 5).

The educational queries presented in Table 6 are based on the educational results and expert opinions.

## 4. Discussion

In this study, we developed a framework for NF2F training of BLS skills, which is indispensable during the COVID-19 pandemic. Through a Delphi survey of experts, an NF2F course was developed and verified through a simulation study. The participation rate of the NF2F group was lower than that of the F2F group in terms of call for help, checks for breathing, and AED operations, which showed similar results in the performance test. By collecting additional opinions from experts after the simulation study, it was confirmed that reinforcing theoretical education through repetitive learning, use of smart devices, and practice is necessary.

Although teachers are crucial to the success of education [17], NF2F training in BLS is very important for several reasons. First, F2F training has limitations owing to the lack of instructors and equipment and the high cost of organizing the curriculum [18,19,20]. Second, NF2F is conducive to the standardization of education, and the instructor’s BLS teaching skills are guaranteed [18]. Third, flexibility in the place and time of the trainees is guaranteed [21]. In addition, NF2F training is more suitable than F2F training in certain situations, such as during the COVID-19 pandemic.

In terms of theoretical knowledge transfer, online education is effective and can partially replace F2F education [22,23]. However, when it comes to teaching practical skills, online training appears to be generally less effective than onsite training [24,25]. Specialist materials and equipment are required in practical training, and it is important to frequently practice [26]. In BLS training, equipment, such as a mannequin on which chest compressions can be performed or an AED can be directly attached, is required. Virtual reality-based BLS training equipment can also facilitate NF2F training if the equipment is unavailable [27]. In our study, although mannequins and AEDs were provided to the trainees, the performance of the NF2F group was lower than that of the F2F group, highlighting the limitation of online education. This can be overcome by reinforcing theoretical education through repetitive learning.

Jensen et al. reported that NF2F training through e-learning is an effective way to maintain advanced life support competence for doctors who have undergone F2F training [28]. The participants’ characteristics in our study were similar to those in the study by Jensen et al., as all participants had previously received BLS training. However, the results were different; BLS competence in the group of participants who underwent F2F training was higher than that in participants who underwent NF2F training in most of the evaluation items. This difference between the studies could be attributed to two factors. First, it may be attributed to differences in the occupations of the study participants. Participants in our study were high-school students, whereas, in the study by Jensen et al., the participants were doctors. Given the nature of medical professions, which require doctors to undergo essential CPR training [29], it is plausible to hypothesize that their participation in and focus on education may have been comparatively higher than that of high-school students. Second, there was a difference in training frequency. In the study by Jensen et al., e-learning was conducted every month for 1 year, whereas, in the present study, education was conducted only once. If we had repeated the training in our study, the NF2F training would have likely produced better CPR performance than that observed using the present methods.

Feedback is essential in education because it has motivating and informative properties; it should include information about knowledge of results (outcomes) or knowledge of performance (skills or movement) [30]. In BLS training, the latter is more applicable. Real-time instructor feedback is important when learning practical skills in NF2F training. However, as there may not be an instructor available to provide feedback, an alternative feedback device can facilitate the acquisition of BLS skills without the support of a professional trainer [31]. Recently, feedback equipment, such as a CPR tutor using a machine learning model, has been developed, which can facilitate NF2F training [32]. The experts in our study also agreed that the use of feedback equipment is essential for NF2F training.

In NF2F education, such as e-learning, it is essential to provide education that effectively covers both theoretical knowledge and practical skills [33]. Theoretical content should be provided in a way that enables learners to gain a thorough understanding of the subject and actively engage in their learning process. Furthermore, assignments or self-testing assessments can be utilized [34]. Regarding practical skills, it is necessary to provide performance-based educational materials that offer a sense of real-world relevance. Evaluation and feedback are crucial to assess whether learners have correctly acquired skills and provide guidance on proper technique [35]. With recent advancements in virtual and augmented reality technologies, educational programs utilizing virtual reality for CPR training have been developed, and their effectiveness has been demonstrated [36,37]. One advantage of utilizing virtual reality for education is that it can enhance the sense of realism and immersion.

Our study had some limitations. First, as the study was conducted with high-school students, caution is needed in generalizing the results. Second, in NF2F training, it is difficult to provide equipment, such as a mannequin. However, the effectiveness of CPR training using equipment, such as virtual reality, has been demonstrated [27]. Third, to bridge the gap between F2F and NF2F education, it is necessary to consider the possibility of distant feedback from instructors. Fourth, the choice of the expert panel is a potential limitation in Delphi studies, as it may be influenced and biased by the researchers’ own sphere of contact. Finally, there are no universally accepted or evidence-based criteria for defining a consensus.

## 5. Conclusions

When F2F training of BLS is not feasible, teach-to-learn innovation is needed, and NF2F training may be a valid option for training CPR knowledge and skills. However, repetitive training, use of assistive devices, and intuitive feedback are crucial aspects of effective NF2F training.

## Figures and Tables

**Table 1:** Demographic characteristics of expert panel.

	Experts N = 13
Age (years)	48 (43–75)
Sex, male	9 (69.2)
Instructor career (years)	15 (11.5–22.5)
Attendance of BLS training sessions in the recent 2 years	30 (10–45)

BLS, basic life support.

**Table 2:** Draft curriculum of basic life support for layperson.

Theoretical Session
Concept of chain of survival
Legal protection for first-aiders
Importance of bystander cardiopulmonary resuscitation
How to recognize a patient in cardiac arrest
**Hands-on skills session**
How to activate emergency medical services system and dispatch cardiopulmonary resuscitation
High-quality chest compressions
Use of automatic external defibrillators
Integrated practice
Evaluation with a feedback device

**Table 3:** General characteristics of participants.

	NF2F Learning N = 35	F2F Learning N = 35	*p*-Value
Sex, male	10 (28.6)	5 (14.3)	0.244
Age (years)	16 (15–17)	17 (16–17)	0.539
Previous BLS training	35 (100)	35 (100)	1.000

BLS, basic life support; F2F, face-to-face; NF2F, non-face-to-face.

**Table 4:** Comparison of class participation and post-training evaluation in NF2F and F2F learning.

	Class Participation			Performance Test		
	NF2F Learning	F2F Learning	*p*-Value	NF2F Learning	F2F Learning	*p*-Value
Assessment and activation						
Checks for responsiveness	35 (100.0)	35 (100.0)	1.000	34 (97.1)	35 (100.0)	1.000
Call for help/AED	11 (31.4)	34 (97.1)	<0.001	29 (82.9)	35 (100.0)	0.025
Points out a helper	12 (34.3)	32 (91.4)	<0.001	24 (68.6)	35 (100.0)	<0.001
Checks breathing	11 (31.4)	35 (100.0)	<0.001	10 (28.6)	30 (85.7)	<0.001
Chest compression						
Chest compression participation rate during 2 min (%)	90.0 (85.8–91.7)	97.5 (95.0–99.2)	<0.001			
High-quality chest compressions				33 (94.3)	34 (97.1)	1.000
Apply AED						
Power on AED	35 (100.0)	35 (100.0)	1.000	31 (88.6)	35 (100.0)	0.114
Correctly attaches pads	32 (91.4)	35 (100.0)	0.239	26 (74.3)	34 (97.1)	0.013
Call out to stand back for analysis	7 (20.0)	33 (94.3)	<0.001	10 (28.6)	32 (91.4)	<0.001
Gesture to stand back for analysis	10 (28.6)	32 (91.4)	<0.001	13 (37.1)	30 (85.7)	<0.001
Call out to stand back for shock delivery	7 (20.0)	32 (91.4)	<0.001	7 (20.0)	34 (97.1)	<0.001
Gesture to stand back for shock delivery	13 (37.1)	29 (82.9)	<0.001	10 (28.6)	31 (88.6)	<0.001
Resume compressions immediately	24 (68.6)	35 (100.0)	<0.001	32 (91.4)	35 (100.0)	0.239

AED, automated external defibrillator; F2F, face-to-face; NF2F, non-face-to-face.

**Table 5:** Suggestion for improvement on items with low performance achievement rate.

Training Content	Suggestion for Improvement
Call for help/AED	Iterative learning Enhanced feedback function using artificial intelligence and smart devices
Call out and gesture to use AED	Iterative learning Reinforcing theoretical session for safety Utilization of auxiliary equipment, such as virtual reality and mobile application
Use of feedback devices	Using intuitive feedback equipment accessible to learners

AED, automatic external defibrillator.

**Table 6:** Suggested curriculum of non-face-to-face basic life support training for laypersons.

Skill That Should Be Trained	Consideration
Theoretical session	
Concept of chain of survival	
Legal protection for first-aiders	
Importance of bystander cardiopulmonary resuscitation	Emphasis on rescuer safety
How to recognize a patient in cardiac arrest and activate emergency medical services system	Emphasis on fully checking breathing for at least 5 s
Hands-on skills session	
How to activate emergency medical services system and dispatch cardiopulmonary resuscitation	Iterative learning
High-quality chest compressions	If real-time interaction with instructor is not available, leverage intuitive feedback using sound or visual guidance
Use of automated external defibrillators	Iterative learning by steps of usage Utilization of auxiliary equipment, such as virtual reality and mobile application
Integrated practice	
Evaluation with a feedback device	Use quantified indicators to compare performance improvement

## Data Availability

The data that support the findings of this study are not openly available due to reasons of sensitivity and are available from the corresponding author upon reasonable request.

## Supplementary Materials

The following supporting information can be downloaded at: https://www.mdpi.com/article/10.3390/healthcare11142110/s1.
